# Frontiers of cosmopolitanism: Educational enclaves and the extractive roots of international schools

**DOI:** 10.1177/0308275X211059655

**Published:** 2021-11-11

**Authors:** Matthieu Bolay, Jeanne Rey

**Affiliations:** 30525 The Graduate Institute of International and Development Studies, Geneva, Switzerland; University of Teachers Education, Fribourg, Switzerland

**Keywords:** Cosmopolitanism, enclaves, extractive industries, international schools, modularity

## Abstract

This article situates international expatriate schools in their cultural and political economy by drawing attention to the tensions between a cosmopolitan educational ethos and processes of social, economic and legal enclavement. Based on extensive multi-sited ethnographic research in the international school sector, we show how cosmopolitan claims of openness mirror a relative closure and ‘offshore-like’ enclavement. To do so, we build upon the notions of modularity and extractivism, which we use as heuristics to analyse social and spatial practices of defining boundaries. Gazing beyond the main foundational myth of international schools, we first outline their concomitant extractive roots. Second, we shed light on the conditions of international teachers’ circulation worldwide. Third, we examine the territorial entanglements and disentanglements that characterise international schools. Finally, we investigate the tensions induced by a cosmopolitan educational ethos whose discourse of inclusion is inevitably paired with practices of exclusion.

Anthropologists have long studied foundational myths, as they are instrumental in shedding light on the various identification processes and boundary-making practices enacted within social groups. In the field of international schooling, whose epistemic community beliefs and group-making practices are the focus of this article, two competing narratives are generally used to explain the origin and global spread of international schools – understood here as schools that provide an international and portable curriculum geared towards global citizenship (see Hughes, 2020: 183–6). Notwithstanding the possibility of a far more complex genealogy behind the concept of the international school (see Sylvester, 2002), the first main narrative ascribes a prominent role to the pedagogical objectives of international schools and locates their birthplace in Switzerland as an outgrowth of the League of Nations (Hill, 2001). The second foundational myth views schooling abroad through a functional lens and situates the origin of the schools in the context of the infrastructure built for Dutch mining corporation Shell’s first oil extraction operations in Malaysia (Brummitt and Keeling, 2013). Currently, international schools are construed mainly according to the League of Nations myth; as such, they are viewed as a pedagogical project rooted in internationalist, pacifist or cosmopolitan endeavours (Fabian, 2016), which sociological analyses have shown to overlap with elitist and class-based strategies geared to the acquisition of international (Wagner, 1998) or cosmopolitan (Weenink, 2008) capital. Yet the innate economic and social environment of the extractive industries, in which many of these schools not only emerged historically but in which they have continued to develop, has been largely overlooked. As such, the League of Nations foundational myth exemplifying the cosmopolitan pole of international schools largely dominates the literature, while the Shell myth and its alternative extractive pole tends to be neglected.

In this article, we draw on the historical linkages between international schools and extractive industries to propose an alternative reading of the development and educational trajectories of these schools. In doing so, however, we do not seek to replace the narrative rooted in educational internationalism with one stemming from expatriation policies of oil and mining companies. We acknowledge that the origins of and causes for the global rise in international education are manifold. For instance, the historical expansion of international curricula has been well documented as lying at the crossroads between idealist and pedagogical projects and coupled with the pragmatic requirements of increasingly mobile upper-class families (Hayden and Thompson, 2013; Hill, 2007: 27; Tarc, 2009b). The contemporary growth of international curricula has also been convincingly explained as a process of distinction in the context of globalisation and neoliberalisation of educational policies (Bolay and Rey, 2020; Doherty et al., 2012; Resnik, 2012), notably in the Global South (Gardner-McTaggart, 2016). This article contributes to this line of scholarship that highlights how international schools are structurally embedded in processes of economic globalisation. We argue that not only is the alternative foundational myth of the Shell school deserving of more scholarly attention, but that there are significant continuities between the continued expansion of international schools and the spreading logistics and spaces of circulation underpinning global extraction supply chains (Watts, 2019). This analytical lens is justified, as the innate spatiotemporal dynamics of extractive industries render the enclave dimension of international schools especially salient. Through this approach we can, first, shed light on the conditions of international teachers’ circulation worldwide; second, examine the territorial entanglements and disentanglements that characterise international schools; and, third, investigate the tensions induced by a cosmopolitan educational ethos whose discourse of inclusion is inevitably paired with practices of exclusion.

We thus scrutinise international schools in contexts of the extraction sector to highlight territorial and educational dynamics that are more broadly relevant for so-called ‘traditional international schools’ catering to expatriate families (Hayden and Thompson, 2013: 5), including schools outside the extractive sector. Indeed, as Junka-Aikio and Cortes-Severino recall (2017: 176–9), ‘extractivism is both a practice [i.e. the literal extraction of minerals] and an ideology [i.e. the existence and reproducibility of new frontiers which capital has to transform into resources]’ that spreads beyond this specific industry. Based on this dual understanding, we follow Mezzadra and Neilson’s call to pay attention ‘to the continuities and ruptures that characterize the relations between *literal extraction* and *extraction in the expanded sense*’ (2017: 188, our italics). We thus question both the *literal* placement of international schools in extractive settings and how *extractivism in the expanded sense* may reverberate in international schools beyond the context of oil and mining projects. We argue that anthropological approaches to extractive enclaves (Appel, 2019; Ferguson, 2005) offer a fruitful set of concepts to address some of the major paradoxes of international schools. In particular, we draw on Appel’s (2012, 2019) conceptualisation of modularity, which articulates the dynamics of territorial entanglement and disentanglement underlying offshore oil extraction. In this article, the lens of modularity is transposed to international schools, and we use it as a heuristic to analyse social and spatial practices of defining boundaries. In these practices, cosmopolitan claims of ‘openness towards divergent cultural experiences’ (Hannerz, 1990: 238) made in international schools mirror the relative closure and ‘offshore-like’ (Ferguson, 2006: 203) geography that characterises the enclaves of extractive projects. In line with the extractive projects, around which many international schools have historically emerged and which continue to be an important context of development for this type of schooling, we contend that these educational cosmopolitan spaces perpetuate themselves by consistently focusing on the maintenance of their own boundaries and their connection to similar enclaved spaces.

Our study draws on ethnographic fieldwork conducted between 2015 and 2018 in 21 international schools located in Switzerland as well as 4 other international schools in North America (Toronto and Chicago). Field research involved either short visits or immersion stays lasting several weeks or months (in six schools). We also attended job fairs (for prospective teachers) in London and Toronto and school fairs (for prospective parents) where dozens of international schools marketed themselves to attract teachers and students. In addition, we conducted 86 interviews with teachers we met in person (in Switzerland, Canada, USA, Kenya and the UK) and online (teachers located worldwide).^1^ For this article, we also consulted internet and media resources such as oil industry blogs and websites, and we mobilised long-standing ethnographic research on extractive industries.

The article begins with a brief conceptualisation of international expatriate schools as modular enclaves. Then, after clarifying the official and alternative myths of origin and their respective linkages to cosmopolitan and extractive epistemologies, the first section focuses on the continuities between the extraction sector and international schools. We build on the case of Shell’s Piasau school in Malaysia to draw a parallel between the spatial-temporal order of extractive enclaves and the trajectories of international schools. From there, we expand on how international teachers experience this form of schooling and show how their adherence to cosmopolitan ideals facilitates their professional mobility across enclaves worldwide. In the second part, we interrogate the continuities between international schools and ‘extraction in an expanded sense’, thereby expanding the concept of modularity to other expatriate international schools. In doing so, we address the work of entanglement and disentanglement that mediates the relations between these schools and their local contexts, namely the architectural, legal and ideological dis/entanglements required to make international schools modular.

## International expatriate schools as modular enclaves

As suggested by the alternative myth of Shell’s Piasau school in Malaysia, the context of multinational extraction companies and commodity trading has played a significant role in the expansion of transnational educational institutions. In Switzerland, where part of the fieldwork was conducted, the explosion of international schools^2^ over the past 20 years has gone hand in hand with an increase in the number of multinational corporations that have established their domicile in the country. For instance, the five major businesses in terms of revenue are extractive and commodity trading companies (Glencore, Vitol, Trafigura, Cargill and Mercuria) and a large percentage of their staff are expatriates. These businesses have specific requirements regarding employee mobility and consequently regarding the schooling of their employees’ children. Moreover, in addition to nurturing corporate culture and values (Garsten, 2003), multinationals have developed specific strategies and models allowing them to situate their projects in the various national and social contexts in which they operate. This is especially salient for oil and mineral supply chains, whose reliance on enclaved modes of operation makes use of what Appel (2019: 22–8) terms modular work of entanglement and disentanglement. As we argue, modular techniques designed to facilitate the circulation of capital, knowledge and resources ‘as if untouched’ (2019: 22) by their direct environment, reverberate strongly in the field of international schools.

Appel proposes the notion of modularity to analyse ‘the practices that bring the offshore into being’ (2012: 693). She suggests that it is the modular features of oil extraction projects that enable offshore work in Equatorial Guinea to function ‘just like offshore work in Ghana, Brazil, or the North Sea’ (2012: 693), or – we would add – in Malaysia. Modularity is thus defined as a ‘work toward disentanglement, including the use of mobile, compliant, and self-contained infrastructures, labor setups, forms of expertise, and legal guidelines’ (Appel, 2012: 693). Nevertheless, Appel (2019: 22) points out that projects of disentanglement from the immediate social and legal environment inevitably generate friction because, more than any other enterprise, extractive operations must touch actual ground. The corporate ideals of disentanglement on which enclaves are constructed require selective legal and political entanglements with the local and national contexts. In the extractive industries, such entanglements are generally performed through ‘local content’ policies (e.g. local procurement or subcontracting) designed to increase national participation in the supply chain. Mobile models of corporate social responsibility (CSR) are another good illustration of how tensions between the global and local settings are addressed in extractive industries, as they rely on continuous ‘processes of abstraction and recontextualization’ (Rajak, 2011: 7) in which corporate actors appeal to assumed universal values that must be embedded in local frameworks and engaged with there. With regard to international schools, Tarc (2009b: 251) makes a similar point, highlighting the ‘operational tension [due to] the universal-particular dynamic of how a standardized [international] curriculum responds to local needs’. As is common with logistics of ‘BYOI’ (Bring you Own Infrastructure) in the extractives (Appel, 2019: 94), modularity thus designates the dual work of entanglement and disentanglement required to globally implement abstract models of extraction – and of education, too, as we argue – by recontextualising them in extremely different local set-ups. The resulting enclaves ultimately enable operations, both extractive and educational, to function as if their local contexts were identical by means of selective separation from the immediate environment.

## An alternative myth: The extractive roots of international schools

As stated earlier, two competing myths of origin are used to explain the emergence of international schools. The most widely circulated narrative situates the origins in 1924, when the ‘Ecole Internationale de Genève’ (Ecolint) first opened. The establishment of the school is generally portrayed as being located at the conjunction of two main endeavours: first, as an initiative of civil servants’ families in Geneva and New York wishing to provide their children with a portable curriculum and to promote peace and intercultural understanding across borders (Hill, 2001); second, as an impulse of Swiss educational reformers, whose progressive pedagogical approaches were spreading across international networks (Sylvester 2002). The narrative of pacifist ideals allied with pedagogical reforms was institutionalised through the launch of the International Baccalaureate Diploma (IB) in 1968. The IB was inspired by the objectives of the United Nations (UN) (Fabian, 2016) and became a major factor in the development of international schools and their continued expansion.

Beyond this narrative, several authors have indicated that the roots of this expanding phenomenon are manifold and include aspects such as US industrial corporate interests promoting post-war economic liberalism (Dugonjić, 2014); pragmatic concerns by an increasingly mobile elite population (Tarc, 2009a); upper-class strategies to maintain a social advantages (Wagner, 1998); middle-class aspirations to acquire social and cultural ‘cosmopolitan’ capital for upward social mobility (Weenink, 2008); and, more broadly, parents’ search for scholastic distinction in times of neoliberal reforms of the public sector (Doherty et al., 2012). In the absence of an official consensus on what makes a school ‘international’ (Hughes, 2020), many schools merely claim the label without formal accreditation through an international curriculum. Nevertheless, in spite of their differences, most international schools share the use of English as a language of instruction and the promotion of ‘international-mindedness’ – though curricula are in fact rather ‘western’ (Bailey, 2021) – as a core feature of their programmes to further the promised development of a cosmopolitan sense of global citizenship beyond the nation-state framework.

There is, however, another myth about the origins of international schools circulating in global recruitment platforms. According to Brummitt and Keeling (2013: 26–7), the first international school was not opened in Geneva but rather in Piasau, near Miri (Malaysia), in 1922 by Dutch mining corporation Shell – two years earlier than the Ecolint school. The following section discusses this (alternative) foundational myth.

## The enmeshed trajectories of an oil deposit and the ‘first international school’

As Shell’s head of Education Services explained in a public interview in 2012 (Mainwaring, 2012), the Piasau school – named after the company employee camp – was an outgrowth of the corporation’s need to respond to a key concern for oil workers: how to maintain family life, including access to education, even in remote locations such as the Malaysian oil fields. After Shell’s first school opened in 1922, other oil and gas companies, including British Petroleum (Shafiee 2018: 101–2), began providing similar services to ensure that workers would be satisfied with the quality of their family life abroad. Similarly, ExxonMobil currently sponsors several schools (and holds positions in their governing body) operated by ISS (International School Services) in countries where the company conducts extraction projects, for instance, in Angola, Nigeria, Indonesia or Kazakhstan. Unlike Ecolint in Geneva, which is still active, the Shell Piasau school closed in 2011, when the oil project was discontinued. Shell moved the school closer to other extractive operations in Sarawak, Malaysia, and renamed it Tenby International School Sarawak. In line with the cosmopolitan ideals of international education, the new school now claims on its website to ‘encourage cultural diversity, tolerance and respect’ and to ‘prepare students for a competitive and fast-changing world’. While the Tenby school propounds the master narrative of an international education embodying cosmopolitan ideals of peace and mutual understanding across borders and cultures, its trajectory as part of the infrastructure of an extractive enclave is characterised by its physical and institutional separation from its direct environment.

As chronicled in *RigZone* (Mainwaring, 2012), a specialised publication dedicated to work in the oil industry, Shell drilled the first oil well in Malaysia in 1910 and, in the nearby city of Miri, quickly consolidated all the infrastructure required to stabilise its operations, including the school. In parallel, Miri grew progressively until the first oil deposits started depleting and some zones, such as Piasau, were requalified as geotourism areas (Sorkhabi, 2010). Indeed, as in any enterprise of the kind, the continuation of operations is subject to financial viability, which in turn depends on various factors, including fluctuating oil prices, rising costs of extraction and the gradual depletion of the deposits. The development and decline of Miri illustrate what Harvey (2003) terms ‘spatial-temporal fixes’, a concept that complements the classic Marxist perspective on the necessity of capital circulation for capital accumulation by adding a spatial layer. As such, spatial fixes of capital inevitably lead to crises of over-accumulation that occur when there is no new object or project in which surplus value can be reinvested, as was the case when Miri’s deposits began to deplete and the city’s economy started to decline (Say and Iqbal, 2016). To overcome such crises, ‘spatial fixes can be expected to be associated with the redirection of capital flows from one space to another’ (Arrighi, 2004: 529), including new extractive frontiers. For instance, Mitchell’s (2011) account of the expansion of the oil industry during the 20th century illustrates how important it has been for this industry to allow fixes of capital to move, despite limitations due to a temporary geographical immobility. In particular, infrastructures such as pipelines, enclaved plants and compounds enable oil production and circulation, while worker circulation, uncontained by unions, overcomes place-based labour.

As the story of Shell in Malaysia illustrates, our understanding of infrastructures in extractive enclaves should also include schools and the overall provision of education. Building on this expanded understanding of the extractive enclave, the next section explores how temporarily immobile infrastructures, as epitomised by international schools in extractive contexts, are circulated to deal with the ineluctable redirection of capital, with a particular focus on human capital, that is, expatriate teachers.

## A redirectionable workforce: International teachers

In his widely cited article ‘Seeing like an oil company’, James Ferguson (2005) mapped how investments in the oil sector have produced enclaved territorialities that do not merely isolate extraction from their local environment, but that also allow new connections to arise:

The movements of capital cross national borders, but they jump point to point, and huge areas are simply bypassed. […] When capital is invested in spatially segregated mineral-extraction enclaves, the ‘flow’ of capital does not cover the globe, it connects discrete points on it. (Ferguson, 2005: 379)

In the case of the Piasau camp near Miri, although the enclave was disentangled from its direct surroundings, it was nevertheless closely connected to global extraction networks. The peripheral situation of children and teachers in the camp meant that the Piasau school also relied on global connections – those of the ‘transnational space of international education’ (Hayden, 2011) – in the form of its international curriculum and the global mobility of teachers who subscribe to cosmopolitan ideals. Indeed, instead of contradicting the closed-off, isolated feature of the schools, cosmopolitanism as it is promoted and circulated in international education rather facilitates the mobility of teachers and students across international schools in extractive settings and beyond.

Among the many international teachers we met and interviewed, most openly claimed that the appeal of international education indeed lies in the cosmopolitan worldview – spurred by a taste for travel and what Hannerz calls a ‘willingness to engage with the Other’ (1990: 239) – promoted in schools and recruitment agencies. Nevertheless, viewing the industry of international schools through a structural lens, it can be observed that the career trajectories of teachers also map offshore-like geographical trajectories akin to those of extractive frontiers. To provide an example of how spatial-temporal fixes shape the mobility of teachers, we consider the trajectory of Sheila, a French teacher of Moroccan heritage, whom we met at a job fair in London.

Sheila was born to Moroccan parents and values the ‘intercultural sensitivity’ that her immigrant background has granted her. She graduated from a teachers’ college in France before moving to Scotland for love and later obtained an equivalent teaching certificate to teach French within the British curriculum. After gaining initial teaching experience, she found employment in Aberdeen, ‘Scotland’s oil capital’, she explained, where she taught mainly children of Total employees and ‘discovered international education’ in a place she describes as a ‘patchwork of nationalities, cultures and religions, a sort of United Nations’. At this time – in the 2000s – the rise of offshore oil in the North Sea was accompanied by a surge in new international schools in Aberdeen. In addition, long-standing schools sponsored by corporations, for instance, the Total school (French) or Shell school (Dutch), were rebranded ‘international’ to accommodate the greater diversity of their employees, a change that was made along with the adoption of the IB curriculum, which replaced the formerly used French or Dutch national curricula.

After a series of changes in her personal life, Sheila decided to ‘go further international’ and attended an international teaching job fair in London in the hopes of finding a new job abroad. There she received offers from schools in Dubai, Florence, Kampala and Houston – with three of the four possible destinations being oil extraction and business centres. Her job offers were in fact representative of the recruiting schools’ locations more broadly. For instance, at two other job fairs we attended, 29 out of 39 and 33 out of 51 schools were in countries with extraction-based economies such as Nigeria, the UAE, Kuwait, Malaysia, Saudi Arabia, Qatar, Colombia, Egypt, Uzbekistan or Kazakhstan, while the remaining ones were mostly located in offshore-like jurisdictions and business hubs in Switzerland, Singapore, Monaco, Honk Kong, Luxembourg, the Bahamas or London. Sheila opted for the job in Kampala, where she had been told that Total was preparing to hire expatriate employees for its new operations that would soon begin in Lake Albert. In her view, teaching in an African country – which she believed would represent larger socio-cultural differences – would be most valued for future positions in international schools and would thus boost her employability – or what Weenink (2008: 1092) would refer to as her ‘cosmopolitan capital’.

Given the very low degree of regulation in this sector, many teachers’ trajectories are far from being as smooth as Sheila’s, and they are often marked by a high degree of precarity (Bunnell, 2016). Our interviews revealed numerous episodes of unanticipated termination of contracts, resulting in the sudden need for teachers to relocate to another school, often to another country. In this context, the cosmopolitan claim of ‘being at home in the world’ (Brennan, 1997) points not only to the intrinsic value attributed to a work environment that is perceived by teachers as diverse and multicultural, but also to a more ‘pragmatic cosmopolitanism’ (Weenink, 2008: 1096–9) that is notably valued in corporate spheres, where a general readiness to relocate for work is expected (Halsall, 2009).

To understand how this being at home in the world is facilitated in the field of international schools, we now draw on the notion of modularity that characterises these extractive projects. Indeed, from our ethnographic work at international job fairs, it appears that school recruiters highlight a strikingly similar set of advantages, albeit in a variety of configurations, to promote their schools, irrespective of location. The work package, including salary and in-kind benefits, the touristic appeal of the location and the quality of the school infrastructure were repeatedly broadcast to attract prospective teachers. In Sheila’s case, all the schools that offered her a position presented extremely similar features, aside from their location. This, to paraphrase Appel (2012: 693), suggests the ability for schools in Kampala to supposedly function ‘just like’ schools in Houston, Florence or Dubai.

## Modularisation of international schools

From a political economy perspective, international schools are likely to emerge where capital is temporarily fixed and circulated, for instance, in areas where finance, mining, logistics and commodity trading firms are concentrated. Switzerland, which is the major trading hub for metals and crude oil (60% and 35% of worldwide trade respectively) (Lannen et al., 2016), provides a telling example, as it hosts some of the largest international trading and extractive firms’ headquarters or trading offices. These include Glencore, BHP, Rusal or Holcim in the Canton of Zug, where 23% of primary and secondary students are enrolled at international schools,^3^ as well as the Geneva-based companies Trafigura, Vitol, Mercuria, Cargill or Gunvor, which cover most of the international school fees of their employees’ children. This model of corporate-sponsored international schools can also be found in new territories of capital absorption such as China, currently the main market for international schools (Poole, 2021), as well as in the many countries where oil and mineral extraction takes place.

As part of the infrastructure that underpins the circulation of capital across these different spaces, international schools must have a modular design, allowing them to share enough features for teachers and students to navigate movement across them smoothly, while also enabling them to become established in their local environment. In the following sections, we identify three areas of entanglement and disentanglement at stake in making international schools modular: architectural, legal, and ideological dis/entanglements. While the resulting enclave model was initially set up for extraction operations, it became institutionalised as a standard feature and a marker of internationalism beyond such settings. Consequently, although the notion of modularity was originally developed to describe operations of extraction, we use it in an extended sense to address the dis/entanglements which are characteristic of international schools for expatriate children.

## Architectural dis/entanglements

As one of the school directors we met in London stated: ‘Parents want to see the same standards and the same offer wherever they live.’ Indeed, the sameness of the schooling environment regardless of the specific local context is an important selection criterion and one that is visible at international school fairs (for prospective families) as well as at international job fairs (for prospective teachers). Parents are familiar with abstract representations of what an international school should provide and look like. They want their children to have everything they need at school, and they want the school structures to be the same, irrespective of the country in which the school is located. Teachers, too, expect the same standards of infrastructure both at school and at home – housing being often provided by the employer within the school compound – wherever they work. In the presentation of the Kampala school chosen by Sheila during a job fair, the director explained the following to the audience of job-seeking teachers:

We have a bar, a supermarket, sport facilities, all within the school complex. So you can enjoy the local food and local culture if you wish, but you can also get everything you are used to, just like in the UK.

For the schools, this implies mainly two aspects of architectural design that are comparable to what Murray (2017) defines as being characteristic of an ‘urbanism of exception’. On the one hand, it means that the school must be physically separated from its immediate environment, and on the other, it indicates the ability to meet everyday needs within the perimeters of the enclave. These aims are achieved by various means, including through specialised architectural firms that develop replicable school designs, thereby leading to an aesthetic that, beyond pursuing functionality, spreads an homogeneous image of what an international school looks like and informs the way clients and staff perceive the ‘global’.

Physical separation then follows a logic of ‘immunity’ that, according to Donner, 2011, characterises extractive enclaves. This logic is meant to liberate residents of the enclaves from the conditions of their immediate environment, which tend to be perceived as a threat to their well-being. Immunity is sought through protective measures that physically isolate schools from the outside world. In many schools we visited or that were presented to us at a job fair, in addition to praising the surrounding fences and walls, the management team would also boast about being equipped with additional protection devices suited to the specific environment of the school. While such infrastructure is common in mining facilities, whose operations and inherent logics of dispossession tend to generate conflict (see Frederiksen and Himley, 2020), schools sometimes resort to security measures in even more absolute ways by constructing the exterior world as a threat, akin to frontier thinking and notions of a menacing wilderness reminiscent of settler colonialism (Veracini, 2010). A school’s immunity materialises in, for instance, the form of security gates, bulletproof windows or roadblocks. And, as an extreme measure, some schools seek to prevent contact with the bio-environment, such as in China, where it has become increasingly popular to cover school campuses with fabric domes that literally insulate the outer limits of the schools and prevent students and staff from breathing polluted air.

What all these measures have in common is that they belong to a set of tools, including architectural and bioprotective systems, that serve to replicate an abstract and standardised model of the international school in any possible location so as to meet so-called ‘international standards’. An additional common feature among all the efforts deployed to realise a shared vision of sameness across schools worldwide is that their inevitable physical location in a national and local territory requires continual legal work to guarantee operations while nonetheless preserving selective forms of disentanglement.

## Legal dis/entanglements

While schooling is historically a core prerogative of the state and a central mechanism of nation-building, most traditional international schools nowadays have their roots in the corporate world and rightly disavow any claim of being the offshoot of a national project. Consequently, schools often have to negotiate their legal relationship with the ‘host country’ – a term ironically inherited from the vocabulary of international investment law that regulates the relationship between states and foreign companies, most notably mining corporations (Burnett and Bret, 2017) – with regard to its regulations on matters of education, but also, as private entities, concerning labour or tax laws. Such relationships presuppose that schools are generally granted a series of legal exceptions, which, according to Barkan (2013), is the underlying principle of ‘corporate sovereignty’, which consists ‘in establishing the legally defined borders and limits of law as well as spaces and cases where laws apply only to the extent of not being applicable’ (2013: 8).

The provision of a non-national curriculum by international schools repeatedly constitutes one such legal exception vis-à-vis the control of school curricula that states have historically exercised to shape the nation’s future citizens and workers (Tomlinson, 2013). In some countries, this question is framed in terms of defining the conditions for accessing an international school, which can range from open access for all students to limited access for certain categories of students, for example, foreigners, bi-nationals or nationals with an immigrant background. In some Swiss cantons, for instance, Zurich, international schools are not open to the local population – regardless of nationality – meaning that only children of expatriate families, whose schooling fees are usually paid by the employer and who can prove that their presence will be of limited duration (e.g. through their work permit), can enrol. In China, there are legal distinctions between international schools for the children of foreign workers, Chinese-owned international schools, and public schools with an international student body parallel to Chinese students (see Assa-Inbar in this issue). These variations translate into divergent conditions of access that separate nationals from foreigners and bi-nationals as well as into divergent curriculum requirements. Whereas typical expatriate schools have no constraints regarding the curriculum they provide, Chinese-owned private schools face strong restrictions on curriculum content and distinguish themselves merely on account of a bilingual English-Chinese teaching context.

Legal exceptions, however, are not restricted to curricular issues: foreign teachers, too, are often granted special status, allowing them to circumvent national regulations governing the teaching labour market. In China, for instance, where the teaching profession is otherwise reserved for Chinese nationals, teachers hired by international schools can obtain a work visa (Z visa), provided that they are native English speakers and have a teaching qualification as well as two years of experience. As we observed, global recruitment platforms act as an intermediary to facilitate these administrative processes.

## Ideological dis/entanglements

The enclave dynamics described above may appear to be in stark contradiction to the cosmopolitan ideals and so-called ‘international-mindedness’ promoted in international schools and continually evoked in reference to the official foundational myth of Ecolint in Geneva. However, rather than being a contradiction, we suggest that it is precisely these cosmopolitan ideals that enable the articulation of the abstract model of the international school in the highly diverse contexts in which it is replicated and recontextualised. While the insistence on international-mindedness obviously relates to the Ecolint foundational myth, expanded notions of extractivism and frontier thinking also arise in teaching practices, which manifest a more discreet reference to the Shell myth.

In teaching practices, for example, the dominant use of English as a language of instruction is upheld as a necessary means to communicate transculturally on the global stage. Nevertheless, English is also complemented by at least one other language, often the local idiom, which represents a symbolic entanglement with the geographical location of the school. Real or imagined, the relationship between the global and local levels is continuously performed in the schools, thereby evoking an external Other as a backdrop against which cosmopolitanism is constructed. Abstract ideas of ‘the local’ are exemplified in three sets of imagined identities: the individual, alleged ‘multiculturality’ of the student body; the ‘culture of the host country’; and the variety of ‘cultures’ deemed traditional and broadly grouped within the Global South and for which international schools enact interconnectedness through charities and philanthropic engagements.

This ‘intercultural context’ is performed daily in othering practices across the three realms mentioned above: students, host country and traditional cultures. First, schools daily celebrate the alleged diversity of the student body. Children are consistently referred to in reference to their nationality, a practice that becomes evident in everyday tasks such as having students draw pictures of their family under their country’s national flag, locate their country of origin on a map, or wear folkloric clothes on international days. Then, the so-called ‘host country’ is also misrepresented through over-simplification in an essentialised cultural context, notably through tourist excursions organised for children and their families. Finally, most schools are engaged in philanthropic projects that primarily serve to enact a cosmopolitan stance. In some of the most affluent schools, children are given the opportunity to travel to countries that are considered safe, yet poor enough to embody a certain image of the Global South, for instance, Thailand or Tanzania. There, international schools often sponsor development-aid-like projects – for example, constructing a school – in which their students are invited to participate. As we have shown elsewhere (Schubiger et al., 2019), such endeavours are sometimes institutionalised in programme-specific requirements of the IB such as the ‘Creativity, Action, Service’ (CAS) component. This mandatory module trains young students to adopt a CSR-mindset that is akin to justifications in the corporate world about the positive impact of global enterprises on local development.

As Resnik (2012: 256) aptly points out, ‘the dispositions fostered in the IB curriculum are similar to the skills needed to succeed in managerial positions in transnational corporations [that] resemble those in the humanist approach to education long encouraged in the IB’. In particular, the set of boundary-making practices interwoven into teaching practices creates a hybrid picture of the world inspired by both cosmopolitan ideals and an extractivist ideology. While students are trained to feel at home in the world, this world-as-one is also perceived as consisting of future frontiers where extractive visions associated with the ‘Shell myth’ resurface in a school’s narratives and performances. For instance, in a TedX Talk organised in 2017 by a Swiss international school, one scientist gave a presentation on possible future asteroid mining. He began his talk with a reference to the journey of Christopher Columbus:

Maybe we are at a similar inflection point, a moment where we might be opening up a new world that will change the lives of our descendants in a way that is comparable to the way the lives of the descendants of Columbus have been changed.

The scientist then presented asteroid mining to the students as a new economic horizon and a technological-scientific challenge, drawing on colonial terminology in the process: ‘We are going to find these resources and once we do, we open up the opportunity to colonise our new world.’

This talk is an extreme illustration of a conception of the world (and beyond) that consists of a set of endless frontiers waiting to be discovered, conquered, exploited – with the social and environmental damage caused in this process having to be remediated at some future date. Such frontier thinking is in fact quite similar to the CSR-inspired endeavours discussed earlier: they construct distant local populations at best as objects of development; otherwise, they are simply targets of compensation measures enacted at expanding extractive frontiers.

## Conclusion: Situating international schools in their cultural and political economy

By paying attention to an alternative genealogy of international education, one that locates the schools’ roots in extractive industries, we were able to foreground the functional role that a modular form of schooling plays in fostering the mobility of expatriate employees across enclaves at expanding extractive frontiers. Despite the salience of this alternative origin, however, the official myth of the International School of Geneva remains central to understanding the rise of international schools, especially with regard to the ways in which abstract cosmopolitan ideals are recontextualised by means of selective entanglements with the local environment. As such, the articulation of the extractive and cosmopolitan epistemologies represented by each separate foundational myth is what helps to better account for the specificity of international expatriate schools.

It should be stated that we have no intention to posit that international schools have a hidden agenda for training future mining executives. Nevertheless, and while we acknowledge the multiple causes of international schools’ expansion and have highlighted some of the features this type of schooling shares with the extraction sector, our argument stresses how the cosmopolitan project of international schooling draws on techniques of modularity that have long-standing roots in the extraction business. The significance of this historical legacy ultimately appears to align cosmopolitan values with a frontier worldview in a broad sense. As Mezzadra and Neilson (2017: 5) assert: ‘Extraction is not limited to mining and drilling for minerals, oil, and gas’ but also involves other processes of capitalist valorisation by prospecting and extracting value that is ‘already there’ (2017: 6). Banking, lending and housing (e.g. Pereira, 2017), financial investment, so-called ‘frontier markets’ (Gilbert, 2018) or, more recently, platforms and digital economies (Sadowski, 2020) are often interpreted as frontier extraction. Seen from this perspective, it is beyond anecdotal, as we observed in a survey conducted with alumni of one major international school in Switzerland, that one-third of the former students have found employment in economic fields taught at business schools, including management, banking, marketing and finance, while only 2.1% of pupils in the broader Swiss national education system undertake higher education in these fields.^4^

Another aspect the two competing myths on the birth of international schools and their respective epistemologies share is that both narratives continuously play on logics of abstraction and re-contextualisation. The form of cosmopolitanism through which international schools conceive ‘the world as one’ (Uimonen, 2020) fosters a point of view that is manifested and enacted through identities, attitudes and practices that facilitate and provide justifications for global mobility in highly different contexts while nonetheless seeking to minimise the frictions that engagement within such contexts might entail. By contrast, the extractive lens through which the world is viewed as an endless frontier for capital goes hand in hand with the architectural, legal and ideological work that in practice aligns features of international schools with those modular components that business operations require to touch ground for varying periods of time.

While the overall picture outlined here is not meant to provide a single, unequivocal interpretation of the worldwide expansion of international schools, by shedding light on some neglected historical roots and continuities between foundational myths, we seek to provide nuance to, and to further complicate, the official discourse circulated in international schools. In doing so, we hope to contribute to better situating certain international ‘expatriate’ schools in their cultural and political economy by drawing attention to the articulation of, and tensions between, a cosmopolitan educational ethos and the extractive-like enclaves of international schools.

## Notes

1 At the time we conducted the interviews, our interlocutors were teaching in, among other places, Singapore, China, Thailand, the USA, Canada, Kenya, Uganda, Kuwait, Switzerland, Qatar, Tanzania, Malta, Spain, UAE and the UK. The global reach of their personal professional trajectories extended even further.

2 In Switzerland, international schools are mostly what Hayden and Thompson (2013) refer to as ‘traditional international schools’, aimed at expatriate children, as opposed to other types of international schools (‘ideology-driven’ or ‘non-traditional’). This article focuses on ‘traditional’ expatriate international schools.

3 Based on the cantonal statistics (https://www.zg.ch/behoerden/gesundheitsdirektion/statistikfachstelle/themen/bildung/2-volksschule-und-privatschulen) and the size of the student body as declared by registered international schools.

4 Swiss Federal Statistical Office: https://www.bfs.admin.ch/bfs/fr/home/statistiques/education-science/scenarios-systeme-formation/hautes-ecoles-etudiants.assetdetail.15384328.html
